# Resistance to Botrytis cinerea in Solanum lycopersicoides involves widespread transcriptional reprogramming

**DOI:** 10.1186/1471-2164-15-334

**Published:** 2014-05-03

**Authors:** Jonathon E Smith, Bemnet Mengesha, Hua Tang, Tesfaye Mengiste, Burton H Bluhm

**Affiliations:** Department of Plant Pathology, University of Arkansas Division of Agriculture, 217 Plant Sciences, Fayetteville, AR 72701 USA; Department of Botany and Plant Pathology, Purdue University, West Lafayette, IN USA

**Keywords:** Necrotrophic pathogenesis, Botrydial, Phytoalexins

## Abstract

**Background:**

Tomato (*Solanum lycopersicum*), one of the world’s most important vegetable crops, is highly susceptible to necrotrophic fungal pathogens such as *Botrytis cinerea* and *Alternaria solani*. Improving resistance through conventional breeding has been hampered by a shortage of resistant germplasm and difficulties in introgressing resistance into elite germplasm without linkage drag. The goal of this study was to explore natural variation among wild *Solanum* species to identify new sources of resistance to necrotrophic fungi and dissect mechanisms underlying resistance against *B. cinerea*.

**Results:**

Among eight wild species evaluated for resistance against *B. cinerea* and *A. solani*, *S. lycopersicoides* expressed the highest levels of resistance against both pathogens. Resistance against *B. cinerea* manifested as containment of pathogen growth. Through next-generation RNA sequencing and *de novo* assembly of the *S. lycopersicoides* transcriptome, changes in gene expression were analyzed during pathogen infection. In response to *B. cinerea,* differentially expressed transcripts grouped into four categories: genes whose expression rapidly increased then rapidly decreased, genes whose expression rapidly increased and plateaued, genes whose expression continually increased, and genes with decreased expression. Homology-based searches also identified a limited number of highly expressed *B. cinerea* genes. Almost immediately after infection by *B. cinerea*, *S. lycopersicoides* suppressed photosynthesis and metabolic processes involved in growth, energy generation, and response to stimuli, and simultaneously induced various defense-related genes, including pathogenesis-related protein 1 (PR1), a beta-1,3-glucanase (glucanase), and a subtilisin-like protease, indicating a shift in priority towards defense. Moreover, cluster analysis revealed novel, uncharacterized genes that may play roles in defense against necrotrophic fungal pathogens in *S. lycopersicoides*. The expression of orthologous defense-related genes in *S. lycopersicum* after infection with *B. cinerea* revealed differences in the onset and intensity of induction, thus illuminating a potential mechanism explaining the increased susceptibility. Additionally, metabolic pathway analyses identified putative defense-related categories of secondary metabolites.

**Conclusions:**

In sum, this study provided insight into resistance against necrotrophic fungal pathogens in the Solanaceae, as well as novel sequence resources for *S. lycopersicoides*.

**Electronic supplementary material:**

The online version of this article (doi: 10.1186/1471-2164-15-334) contains supplementary material, which is available to authorized users.

## Background

Plant pathogens are classified as necrotrophs, biotrophs, or hemibiotrophs based on their modes of nutrition [1–3]. Biotrophs feed on living tissue and subtly manipulate host physiology to obtain nutrients [1, 2]. Necrotrophs kill host cells to obtain nutrients, often inducing expanding, necrotic lesions [1, 4]. Hemibiotrophs undergo a biotrophic stage of nutrition before shifting to a necrotrophic strategy for nutrient uptake [1, 3]. Due to their fundamentally distinct mechanism of pathogenesis, biotrophs have evolved mechanisms to suppress cell death while necrotrophs promote it as a virulence strategy [5–8]. When hosts fail to constrain necrosis caused by necrotrophs and hemibiotrophs, diseases can culminate in the death and decay of the entire plant. Toxins and hydrolytic enzymes are central to virulence in necrotrophs but have minimal contributions to biotrophic pathogenesis [2, 4, 8]. Consequently, host responses to pathogen infection vary depending on the nature of the pathogen. Whereas the molecular basis of resistance against biotrophic infection strategies is becoming increasingly well understood [9, 10], the current understanding of plant resistance against necrotrophic fungi is fragmentary.

Necrotrophs are classified as either broad host-range or host-specific pathogens [8]. While broad-host-range necrotrophs produce a variety of cell wall-degrading enzymes, phytotoxic metabolites, and cell death elicitors that kill host cells and induce necrosis, the ability of host-specific necrotrophs to cause disease is generally attributed to the production of toxins that have activity on a limited number of related plant species [11, 12]. The broad host-range necrotroph, *Botrytis cinerea*, is a ubiquitous and cosmopolitan pathogen that causes gray mold disease on more than 200 host plants [13] with worldwide losses in affected crops estimated at 20% [14]. *B. cinerea* induces necrosis by producing toxins and reactive oxygen species [15, 16], and also manipulates hosts into producing oxidative bursts that facilitate colonization [17, 18]. Two classes of toxins have been identified in *B. cinerea* that exhibit non-specific phytotoxicity: the sesquiterpene toxin, botrydial, and related metabolites, and the polyketide toxin, botcinic acid, and its derivatives [15, 19–21]. In contrast to *B. cinerea*, *Alternaria solani* primarily infects members of the Solanaceae such as tomato, potato, peppers, and eggplant [22]. Like *B. cinerea*, *A. solani* uses toxins to induce necrosis in its hosts [23]. While as many as eleven toxins have been identified in cultures of *A. solani*, alternaric acid and solanopyrones A, B, and C, have been implicated as the primary necrosis-inducing toxins [22, 24, 25]. Although necrosis of host tissues is known to be induced by toxins, additional, unknown factors may be involved in the host specificity of *A. solani*[24].

The Solanaceae is one of the world’s most economically important plant families and includes vegetables, ornamentals, and medicinal plants [26]. Among the solanaceous crops, tomato (*Solanum lycopersicum*) is particularly susceptible to *B. cinerea* and *A. solani*[27, 28]. Due to a lack of genetic resistance against necrotrophic fungal pathogens in commercial tomato cultivars, *B. cinerea* and *A. solani* inflict heavy losses, and thus frequent applications of fungicides are required for disease management. In the absence of chemical protection, over 50% of the annual tomato crop can be lost to necrotrophic pathogens [29]. Although tomato lacks resistance to *B. cinerea* and *A. solani*, robust resistance against some necrotrophic fungal pathogens has been identified in closely related species within the Solanaceae [30, 31]. However, the underlying mechanisms of resistance have not been characterized at the molecular level, in part due to a lack of molecular resources for many members of the Solanaceae, particularly non-crop species.

Identification and characterization of genetic resistance against necrotrophic fungi would provide a crucial biological foundation for crop improvement within the Solanaceae. The overarching goal of this study was to identify and characterize resistance to necrotrophic fungal pathogens among members of the Solanaceae. To this end, we screened a panel of *Solanum* species for resistance to *B. cinerea* and *A. solani* and found that *S. lycopersicoides* (LA2951) showed a high level of resistance to both pathogens. This resistance manifested as constrained lesion expansion as well as reduced pathogen growth. Then, we generated gene expression profiles from *S. lycopersicoides* 24 and 48 hours after inoculation with *B. cinerea*, as well as a pre-infection baseline, via high-throughput RNA-sequencing (Roche-454). Analyses of the transcriptomes revealed that numerous genes were differentially expressed in *S. lycopersicoides* in response to *B. cinerea*, including pathogenesis-related proteins, proteases, a glucanase, and genes involved in biosynthesis of secondary metabolites. Additionally, a set of highly expressed *B. cinerea* genes was identified, which could facilitate the elucidation of fungal genes involved in necrotrophic pathogenesis.

## Results

### Evaluation of resistance against necrotrophic fungi among wild Solanum species

**Figure 1:**
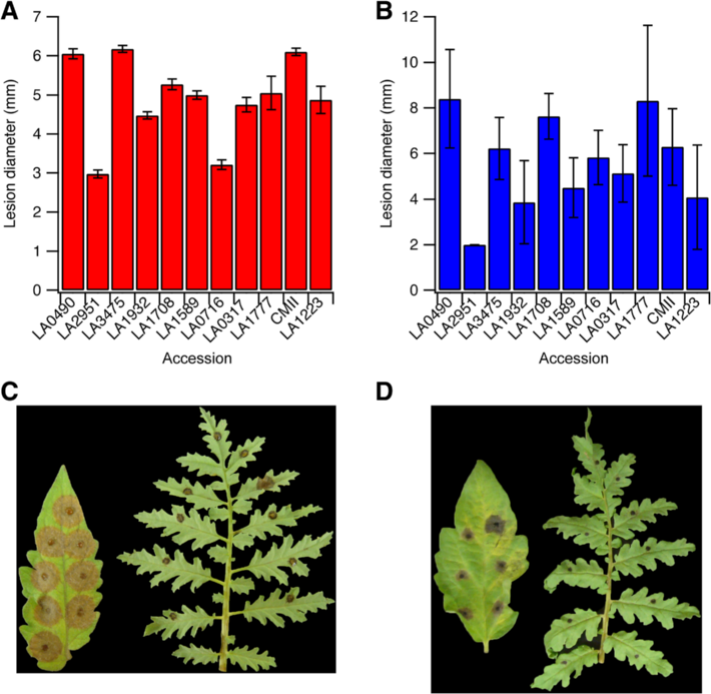
**Comparison of resistance to necrotrophic fungal pathogens among** ***Solanum*** **species.** Quantitative comparison of resistance to **(A)** *B. cinerea* and **(B)** *A. solani* among *Solanum* species. Visual comparison of disease development on *S. lycopersicum* and *S. lycopersicoides* 96 hours after inoculation with *B. cinerea* **(C)** and *A. solani* **(D)**.

Resistance to *B. cinerea* has been previously reported in wild solanaceous plants [30, 32, 33]. However, to date, no study has quantitatively compared resistance to multiple necrotrophic fungal pathogens in *Solanum* species, and it is not known whether mechanisms of resistance to *B. cinerea* are the same for other necrotrophs. Therefore, three tomato varieties and eight wild *Solanum* species were evaluated for resistance against *B. cinerea* and *A. solani* (Figure 1). The tomato varieties VF-36 (*S. lycopersicum*, LA0490) and M-82 (*S. lycopersicum*, LA3475) are parents for introgression lines created from *S. lycopersicoides* and *S. pennellii*, respectively, and were selected as susceptible checks [34, 35]. The variety Castlemart II was selected because of its common usage in tomato genetics studies. All of the wild *Solanum* species selected are native to South America; the center of origin for tomato is the Andean region of South America and thus wild species from this area are more likely to be naturally adapted to challenge by pathogens [36].

Of the eleven lines tested, *Solanum lycopersicoides* (LA2951) was the most resistant to both *B. cinerea* and *A. solani* (Figure 1A, B)*,* suggesting the presence of broad-spectrum resistance to necrotrophs. *B. cinerea* caused indistinguishably high levels of necrosis in all three *S. lycopersicum* varieties tested. In contrast, the wild *Solanum* species showed varying levels of resistance, which manifested as a reduction in lesion diameter compared to the *S. lycopersicum* varieties. The reduction in lesion diameter ranged from 13% for *S. arcanum* (LA1708) to 51% for *S. lycopersicoides* (LA2951)*.* A high level of resistance to *B. cinerea* was also observed in *S. pennellii* (LA0716), which showed a 47% reduction in lesion diameter as compared to *S. lycopersicum*. Interestingly, resistance responses to *A. solani* followed a different pattern than observed for *B. cinerea*. Among the eleven lines tested, VF-36 (*S. lycopersicum*, LA0490) was the most susceptible. The *S. lycopersicum* varieties were not equally susceptible to *A. solani*; M-82 and Castlemart II exhibited a 26% and 25% reduction in lesion diameter respectively as compared to VF-36. Among the wild species tested, *S. lycopersicoides* (LA2951) was the most resistant to *A. solani,* and appeared to exhibit even higher levels of resistance to *A. solani* than *B. cinerea*. In contrast, *S. pennellii* (LA0716) was only moderately resistant to *A. solani* but was highly resistant to *B. cinerea*. Therefore, given the high level of resistance of *S. lycopersicoides* to both necrotrophic pathogens, this accession was selected to investigate molecular mechanisms of resistance to necrotrophs (Figure 1C, D). *B. cinerea* was chosen to serve a model necrotroph in this study because it has a sequenced genome [37], readily sporulates in culture, and causes disease on all tomato varieties tested.

### Characterization of resistance against B. cinerea in S. lycopersicoides

**Figure 2:**
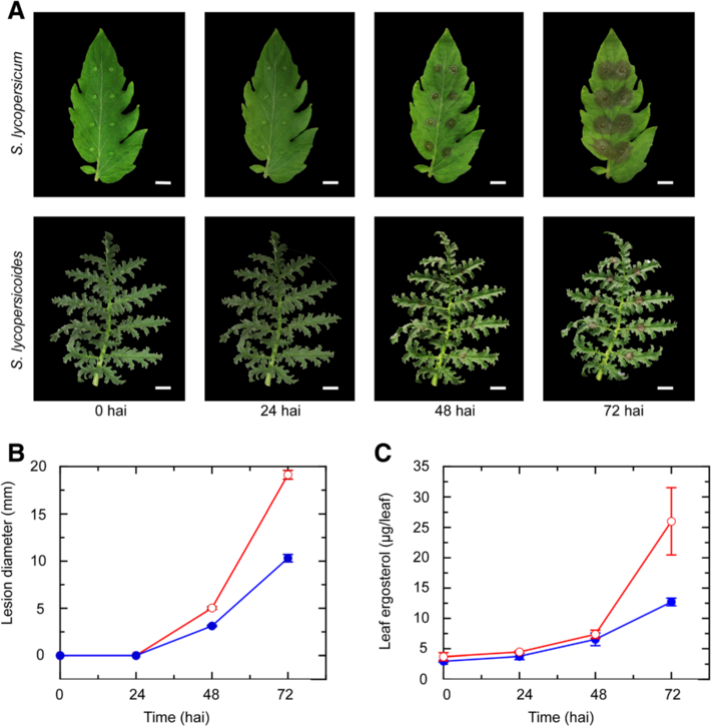
**Comparison of disease development on** ***Solanum lycopersicum*** **and** ***Solanum lycopersicoides***. **(A)** Lesion development on *S. lycopersicum* and *S. lycopersicoides* was observed at 0, 24, 48, and 72 hours after inoculation (hai) with *B. cinerea*. White bars represent 10 mm. **(B)** Lesion diameter and **(C)** ergosterol content were measured to quantify disease development and fungal growth, respectively, on *S. lycopersicoides* (Blue) and *S. lycopersicum* (Red)*.* **(B)** For lesion diameter, values represent the mean diameter of 32 individual lesions, and error bars indicate standard error. **(C)** For ergosterol content, values represent the mean of 3 separate leaves, and error bars indicate standard error. Both fungal growth and lesion diameter increased in *S. lycopersicum* compared to *S. lycopersicoides* at 72 h after inoculation.

To further define resistance against *B. cinerea* in *S. lycopersicoides*, the sequenced wild-type strain of *B. cinerea* (B05.10) was inoculated on *S. lycopersicoides* accession LA2951 (resistant to *B. cinerea*) and *S. lycopersicum* cv. Bradley (susceptible to *B. cinerea*). Symptoms of infection were observed by 48 h after inoculation in both *S. lycopersicum* and *S. lycopersicoides* (Figure 2A), although lesions were 61% larger on *S. lycopersicum* than *S. lycopersicoides* (Figure 2B). Symptoms initially appeared as small, water-soaked lesions that quickly became necrotic and spreading. By 72 h after inoculation, lesions on *S. lycopersicum* began to coalesce. Likewise, due to the leaf morphology of *S. lycopersicoides*, some lesions began to reach the edges of leaflets. However, lesions on *S. lycopersicum* were nearly twice the diameter of those on *S. lycopersicoides* (Figure 2B).

To determine whether the smaller lesions on *S. lycopersicoides* were due primarily to reduced pathogen growth, ergosterol was quantified from *S. lycopersicum* and *S. lycopersicoides* leaves inoculated with *B. cinerea*. Interestingly, fungal growth was not significantly different between *S. lycopersicum* and *S. lycopersicoides* 48 h after inoculation (Figure 2C). However, by 72 h after inoculation, the ergosterol content of *S. lycopersicum* was over twice that of inoculated *S. lycopersicoides* leaves (Figure 2C). The increased detection of *B. cinerea* in *S. lycopersicum* as compared to *S. lycopersicoides* 72 h after inoculation correlated closely with observed levels of necrosis and indicates that suppression of fungal growth may be a primary component of resistance to *B. cinerea* in *S. lycopersicoides.*

### De novo assembly of the S. lycopersicoides transcriptome

Currently, a reference genome sequence is not available for *S. lycopersicoides* and a very limited number of nucleotide sequences are deposited in GenBank for this organism. Thus, to elucidate mechanisms of resistance to necrotrophs and provide a novel sequence resource, 454 pyrosequencing was used to sequence the transcriptome of *S. lycopersicoides* leaves 24 and 48 h after inoculation with *B. cinerea*, as well as leaves collected immediately after inoculation (0 h) to provide a baseline for comparison. A total of 654,159 reads consisting of nearly 165 Mb were obtained (Table 1). Over 550,000 of the reads were assembled into 13,008 contigs, which were then assembled into 11,916 isotigs and 10,255 isogroups. Isogroups corresponded to genes, while isotigs within an isogroup represented splice variants of the gene. To reduce overrepresentation of genes with multiple splice variants, read counts from each isotig within an isogroup were summed. A representative isotig from each isogroup and the remaining contigs that were not assembled into isotigs accounted for a total of 10,385 unigenes.

**Table 1:** **Summary of Roche 454 GS-FLX assembly of** ***S. lycopersicoides*** **transcriptome sequences**

Metric	Sequence (n)	Bases (bp)
Total reads	654,159	164,925,531
Average read length		252
Aligned reads	554,524 (84.77%)	140,923,440 (85.45%)
Average trimmed read length		254
All contigs	13,008	9,327,270
Average contig length		717
Large contigs	8,431	7,863,766
Average large contig length		932
N50 large contig length		988
Largest contig		4,469
Isotigs	11,916	1,0722,599
Average isotig length		899
N50 isotig length		1,041
Largest isotig		4,504

The BLASTx algorithm was used to distinguish unigenes of *S. lycopercicoides* from those of *B. cinerea* and to remove sequences from contaminating species (e.g. bacteria and viruses). Of the 10,385 unigenes, 382 did not match any sequence in the non-redundant protein sequences database (nr, NCBI) or matched contaminating organisms and were thus excluded from further analyses. Of the remaining 10,003 unigenes, 9,414 (94.1%) had significant matches with sequences from plant species and were thus determined to be *S. lyocpersicoides* sequences, whereas 589 (5.9%) were determined to be of fungal origin. Among the 9,414 unigenes determined to be of plant origin, nearly 91% (8,566) were highly similar to genes from *S. lycopersicum*, which has a sequenced reference genome [38], and an additional 5% (466) were highly similar to genes from other species of Solanaceae, including *S. tuberosum*, *Nicotiana tobacum*, and *Capsicum annuum*. The remaining 4% (382) of *S. lycopersicoides* unigenes were most similar to sequences found in comparatively distant plant species, including *A. thaliana*, *Medicago truncatula*, and *Populus trichocarpa*. The high percentage of *S. lycopersicoides* unigenes matching sequences from other members of the Solanaceae validates the *de novo* assembly of the *S. lycopersicoides* transcriptome and indicates high levels of sequence conservation between *S. lycopersicoides* and related species.

### Cluster analyses reveal distinct patterns of gene expression in response to B. cinerea

**Figure 3:**
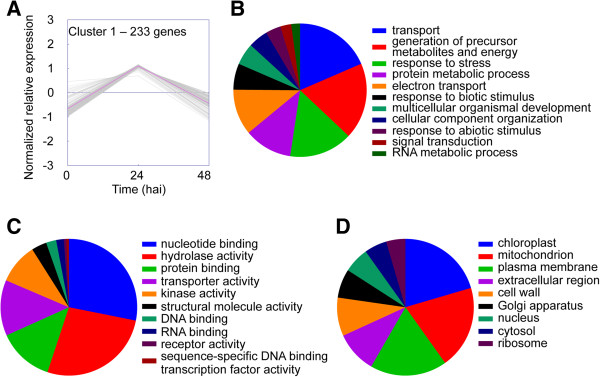
**GO terms associated with unigenes in expression cluster 1. (A)** K-means clustering was used to visualize the expression pattern of unigenes in cluster 1. Expression increased rapidly from 0 to 24 h after inoculation then decreased to basal levels by 48 h after inoculation. Unigenes were annotated with GO terms corresponding to **(B)** biological processes, **(C)** molecular functions, and **(D)** cellular components. Pie charts represent the distribution of unigenes annotated with each GO term.

**Figure 4:**
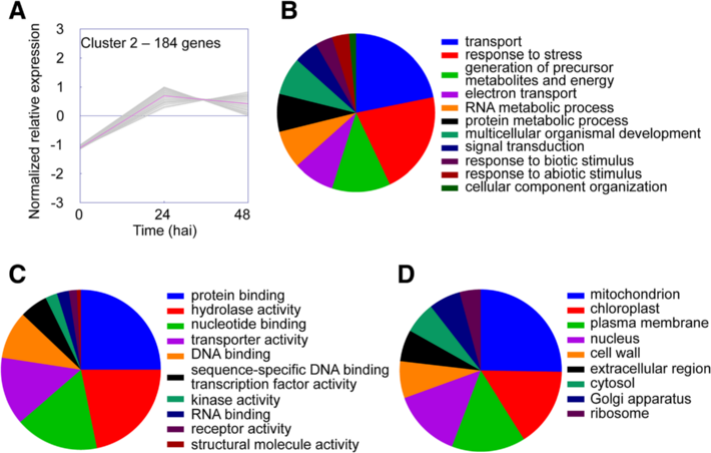
**GO terms associated with unigenes in expression cluster 2. (A)** K-means clustering was used to visualize the expression pattern of unigenes in cluster 2. Expression increased rapidly from 0 to 24 h after inoculation then plateaued. Unigenes were annotated with GO terms corresponding to **(B)** biological processes, **(C)** molecular functions, and **(D)** cellular components. Pie charts represent the distribution of unigenes annotated with each GO term.

**Figure 5:**
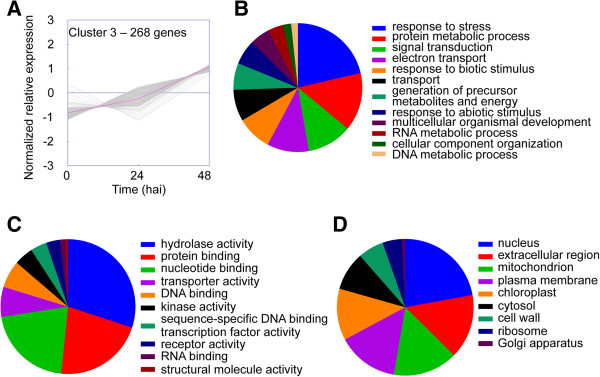
**GO terms associated with unigenes in expression cluster 3. (A)** K-means clustering was used to visualize the expression pattern of unigenes in cluster 3. Expression increased continually from 0 to 48 h after inoculation. Unigenes were annotated with GO terms corresponding to **(B)** biological processes, **(C)** molecular functions, and **(D)** cellular components. Pie charts represent the distribution of unigenes annotated with each GO term.

**Figure 6:**
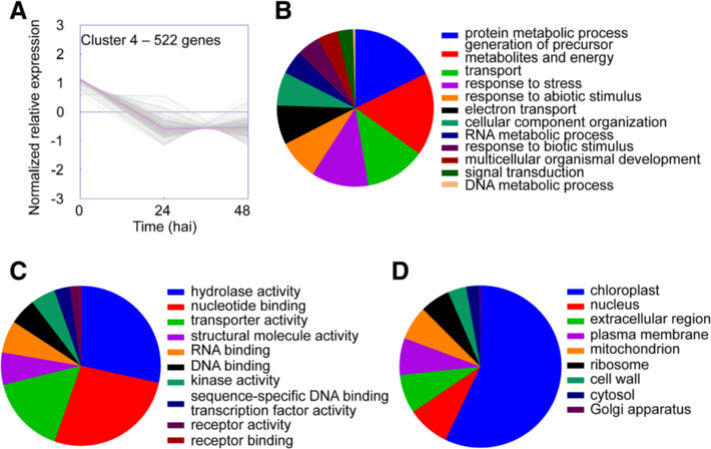
**GO terms associated with unigenes in expression cluster 4. (A)** K-means clustering was used to visualize the expression pattern of unigenes in cluster 4. Expression decreased from 0 to 48 h after inoculation. Unigenes were annotated with GO terms corresponding to **(B)** biological processes, **(C)** molecular functions, and **(D)** cellular components. Pie charts represent the distribution of unigenes annotated with each GO term.

Differentially expressed *S. lycopersicoides* unigenes (Additional file 1) grouped into four distinct clusters. Cluster 1 contained genes induced 24 h after inoculation with decreased expression thereafter (Figure 3). Cluster 2 contained genes induced 24 h after inoculation whose expression remained up-regulated (Figure 4). Cluster 3 represented genes whose expression increased continually throughout the time course (Figure 5), and Cluster 4 represented genes in *S. lycopersicoides* that were down-regulated in response to *B. cinerea* infection (Figure 6). To examine potential relationships between expression pattern and biological function, unigenes in each cluster were assigned gene ontology (GO) terms in three categories: biological processes, molecular functions and cellular components. However, this initial assignment of GO terms was too broad, resulting in hundreds of terms, and thus a GO slim analysis was performed. GO slim is a defined list of high-level GO terms that cover broad aspects of processes, functions, and components [39].

GO slim analyses revealed many similarities between clusters 1 and 2. The major GO slim terms for biological processes associated with cluster 1 were “transport”, “generation of precursor metabolites and energy”, and “response to stress” (Figure 3B), and the major GO slim terms for molecular function were “nucleotide binding” and “hydrolase activity” (Figure 3C). The major GO slim terms for cellular component were “chloroplast”, “mitochondrion”, and “plasma membrane” (Figure 3D). Similar to cluster 1, the major GO slim terms for biological processes associated with cluster 2 were “transport”, “response to stress”, and “generation of precursor metabolites and energy” (Figure 4B), and the major GO slim terms for molecular function were “protein binding”, “hydrolase activity”, and “nucleotide binding” (Figure 4C). The major GO slim terms for cellular component were “mitochondrion”, “chloroplast”, and “plasma membrane” (Figure 4D). The similarities between biological processes, molecular functions, and cellular components for clusters 1 and 2 suggest that these two groups of genes are involved in similar responses to *B. cinerea*.

For cluster 3, GO slim terms were substantially different than clusters 1, 2, or 4. Specifically, the major GO slim terms for biological processes associated with cluster 3 were “response to stress”, “protein metabolic process”, “signal transduction”, and “electron transport” (Figure 5B), and the major GO slim terms for molecular function were “hydrolase activity”, “protein binding”, and “nucleotide binding” (Figure 5C). The major GO slim terms for cellular component were “nucleus”, “extracellular region”, and “mitochondrion” (Figure 5D). The induction of cluster 3 genes after pathogen attack is consistent with induced defense responses, however, the substantial differences in major GO slim terms in cluster 3 as compared to clusters 1 and 2 may reflect distinctly separate mechanisms of defense.

Cluster 4 contained the most pronounced differences in GO slim terms among the four clusters. The major GO slim terms for biological processes associated with this cluster were “protein metabolic process”, “generation of precursor metabolites and energy”, “transport”, and “response to stress” (Figure 6B), and the major GO slim terms for molecular function were “hydrolase activity” and “nucleotide binding” (Figure 6C). The major GO slim term for cellular component was “chloroplast” (Figure 6D). Overall, these results strongly suggest a rapid and intense suppression of primary metabolism upon challenge with *B. cinerea*, presumably due to resource reallocation to defense responses.

**Figure 7:**
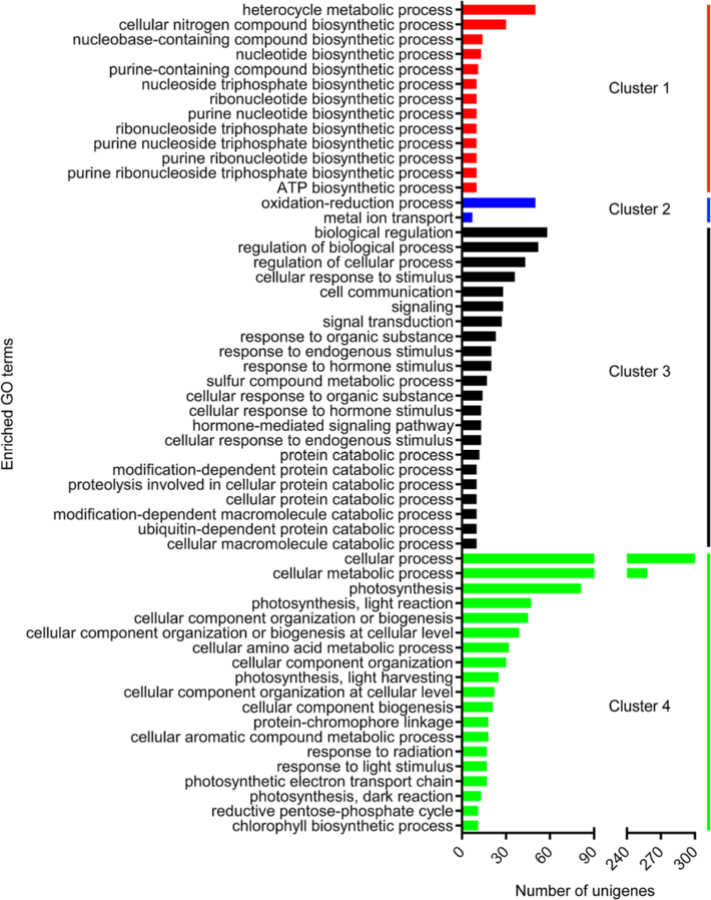
**Enrichment analysis of biological processes associated with each expression cluster.** Enrichment analysis was performed to determine which biological processes were present at significantly higher frequency in cluster 1 (red bars), cluster 2 (blue bars), cluster 3 (black bars), and cluster 4 (green bars). The X-axis indicates the number of unigenes annotated with a given GO term.

To complement the broad overview of the processes, functions, and components provided by GO slim terms, an enrichment analysis identified individual GO terms that were significantly over-represented in each cluster of unigenes (Figure 7). In cluster 1, 13 biological processes were significantly enriched, all of which were associated with the production of ATP. Peak expression of genes involved in ATP biosynthesis at 24 h after inoculation is consistent with the rapid conversion of precursor metabolites to energy upon pathogen attack. Conversely, two terms were enriched in cluster 2, with one unigene assigned to each term. The two GO terms enriched in cluster 2 are broad and somewhat uninformative; “oxidation-reduction process” and “metal ion transport” could be involved in many aspects of metabolism. Additionally, the small number of significantly over-represented GO terms in cluster 2 suggests that unigenes in this cluster share similar overall functional annotation with other clusters. Cluster 3 was enriched with terms involved in “signaling”, “protein degradation”, and “response to stimuli”. Enrichment of the term “signaling” is in agreement with the findings of Windram et al. [40], who showed that genes involved in signaling were rapidly upregulated in Arabidopsis as early as 16 hours after inoculation with *B. cinerea*. Furthermore, De Cremer et al. [41] demonstated that genes involved in signaling were upregulated in lettuce, 48 hours after inoculation with *B. cinerea*. Enrichment of these terms indicates that *S. lycopersicoides* mounts an early and sustained signaling response to infection by *B. cinerea*, as would be expected by the requirement for continual containment of a necrotrophic fungal pathogen. Cluster 4 was significantly enriched with multiple terms relating to photosynthesis, which is consistent with the rapid down-regulation of genes involved in photosynthesis in *B. cinerea* infected plants described by Berger et al. [42], Windram et al. [40], and De Cremer et al. [41]. Taken together, the cluster analyses provided insight into potential linkages between gene expression patterns and biological responses underlying resistance to *B. cinerea* in *S. lycopersicoides*.

### Comparative expression analysis of selected genes in S. lycopersicoides and S. lycopersicum

**Figure 8:**
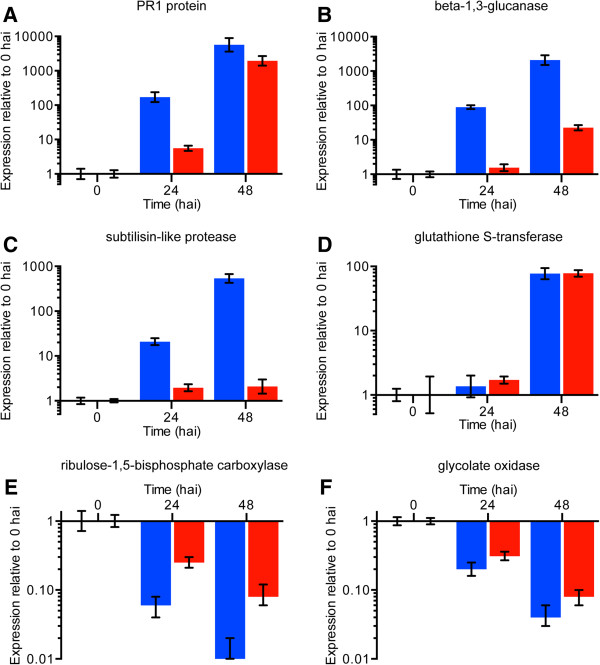
**Relative expression of selected genes measured by qPCR.** Different patterns of gene expression were observed for *S. lycopersicoides* and *S. lycopersicum* inoculated with *B. cinerea*. Induction of **(A)** a PR1 protein-encoding gene was delayed in *S. lycopersicum* compared to *S. lycopersicoides*, but expression at 48 hours after inoculation (hai) was comparable in both *Solanum* species. Induction of **(B)** a beta-1,3-glucanase-encoding gene and **(C)** a subtilisin-like protease-encoding gene was also delayed in *S. lycopersicum*; however, expression at 48 hai was reduced compared to *S. lycopersicoides*. Induction and expression of **(D)** a glutathione S-transferase-encoding gene were comparable between *S. lycopersicum* and *S. lycopersicoides*. Expression of **(E)** a ribulose-1,5-bisphosphate carboxylase-encoding gene and **(F)** a glycolate oxidase-encoding gene decreased over time in both *S. lycopersicum* and *S. lycopersicoides*, but the rate and overall reduction of expression was greater in *S. lycopersicoides*. Genes were selected for qPCR analysis based on expression and predicted role in pathogen defense. Blue bars represent expression of genes in *S. lycopersicoides* relative to timepoint 0. Red bars represent expression of genes in *S. lycopersicum* relative to timepoint 0.

To confirm the RNA-seq data from *S. lycopersicoides* and provide insight into differential transcriptional responses to *B. cinerea* in *S. lycopersicum*, six differentially-expressed genes (induced and suppressed in response to *B. cinerea*) were assessed in *S. lycopersicoides* and *S. lycopersicum* with quantitative PCR (qPCR)*.* Four genes reported to be involved in resistance to necrotrophs (pathogenesis-related protein 1 (PR1) [43, 44], a beta-1,3-glucanase (glucanase) [44], a subtilisin-like protease [45], and glutathione S-transferase [46, 47]) were chosen to represent defense-related genes induced upon attack by *B. cinerea*, and two genes involved in photorespiration (ribulose-1,5-bisphosphate carboxylase (Rubisco) small subunit and glycolate oxidase 1) were chosen to represent down-regulated genes. Upon challenge with *B. cinerea*, potentially important differences in expression profiles of the selected genes were observed in comparisons between *S. lycopersicum* and *S. lycopersicoides.* Among the genes induced during infection by *B. cinerea* in *S. lycopersicoides*, several differences in induction patterns were observed in *S. lycopersicum*. As an example of delayed induction, expression of PR1 rapidly increased in *S. lycopersicoides* from 0 to 24 h after inoculation as observed in the RNA-seq analysis, whereas induction in *S. lycopersicum* was not observed until 48 h after inoculation, at which time expression levels were comparable to *S. lycopersicoides* (Figure 8A). As an example of delayed and reduced induction, expression of glucanase continually increased in response to *B. cinerea* in *S. lycopersicoides* as previously determined through transcriptomics*,* but the induction in tomato was consistently later and at lower levels than observed in *S. lycopersicoides* (Figure 8B). Expression of a subtilisin-like protease in *S. lycopersicum* provided an even more pronounced example of delayed and reduced induction, whereas the induction pattern observed in *S. lycopersicoides* was consistent with results from RNA-seq analyses (Figure 8C). However, at least some genes were similarly induced in *S. lycopersicoides* and *S. lycopersicum* in response to *B. cinerea*, as evidenced by glutathione S-transferase (Figure 8D). Additionally, expression of the genes involved in photorespiration, particularly Rubisco, dropped more dramatically in *S. lycopersicoides* than in *S. lycopersicum* (Figure 8E, F), which suggests that the speed at which photosynthesis is suspended after pathogen attack may play a critical role in defense-related resource reallocation.

### Metabolic pathway analysis

For metabolic pathway mapping, KEGG (Kyoto Encyclopedia of Genes and Genomes) orthology (KO) identifiers were assigned throughout the four differentially expressed clusters of *S. lycopersicoides* genes which were then mapped individually to pathway maps in the KEGG database. This process identified potential shunts in metabolism resulting from *B. cinerea* infection, the most striking example of which was in the pathway for terpenoid backbone biosynthesis (Additional file 2). Specifically, several genes in the 2-C-methyl-D-erythritol 4-phosphate (MEP) pathway were suppressed in response to *B. cinerea*, while genes in the mevalonate pathway were induced. The mevalonate pathway is used by plants for the biosynthesis of sesquiterpene phytoalexins [48–50], while the MEP pathway is localized in plastids and is the pathway for the production of structurally distinct terpenoids including carotenoids and the phytol chain of chlorophyll [50]. Recently, the MEP pathway was also implicated in stress response [51, 52]. The MEP pathway acts as stress sensor and, through the biosynthesis of retrograde signaling molecules, an inducer of stress response genes. However, accumulation of methylerythritol cyclodiphosphate (MEcPP), a stress-induced, retrograde signaling molecule produced via the MEP pathway, is associated with abiotic stress and results in increased resistance to biotrophs and enhanced susceptibility to *B. cinerea*[51]. Thus, the coordinated change in gene expression from the MEP pathway to the mevalonate pathway, in *S. lycopersicoides* during defense against *B. cinerea*, is consistent with a shift away from abiotic stress response and biotrophic pathogen resistance and with the increased phytoalexin biosynthesis observed in other solanaceous plants [48, 53].

### Identification of B. cinerea genes highly expressed during infection

**Figure 9:**
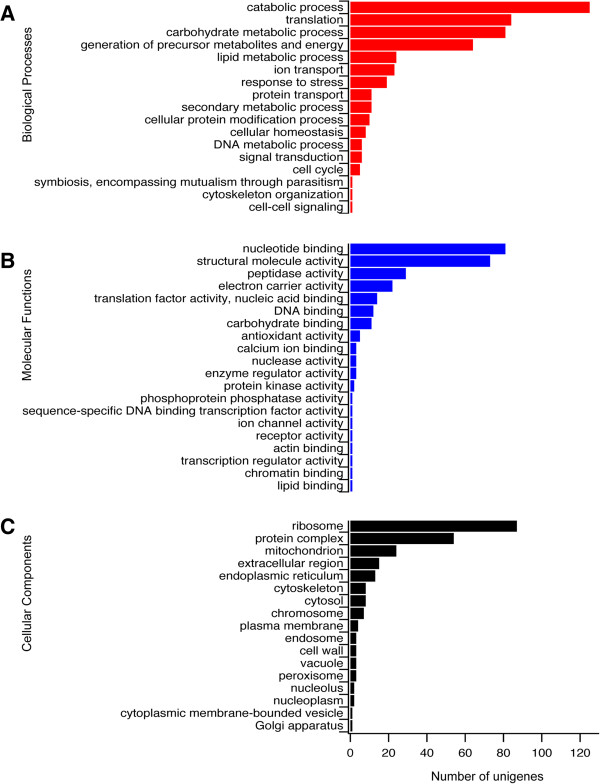
**GO analysis of** ***B. cinerea*** **unigenes expressed during infection of** ***S. lycopersicoides.*** Fungal unigenes were annotated with GO terms corresponding to **(A)** biological processes (red bars), **(B)** molecular functions (blue bars), and **(C)** cellular components (black bars). The X-axis indicates the number of unigenes annotated with a given GO term.

GO slim analyses were performed on the 589 unigenes determined to be of fungal origin (Additional file 3). The biological processes most represented in *B. cinerea* were “catabolic process”, “translation”, “carbohydrate metabolic process”, and “generation of precursor metabolites and energy” (Figure 9A). The abundance of unigenes associated with catabolic processes and carbohydrate metabolic processes is suggestive of the induction of cell wall degrading enzymes, while the presence of numerous unigenes associated with translation and energy generation is consistent with rapid colonization of *S. lycopersicoides* by *B. cinerea*. Furthermore, the term “secondary metabolic process” suggests a prominent role for the biosynthesis of phytotoxic compounds during pathogenesis. The molecular functions associated with *B. cinerea* infecting *S. lycopersicoides* are consistent with the biological processes. The most represented function was “nucleotide binding” (Figure 9B), which suggests widespread and dynamic reprogramming of the fungal transcriptome required to sustain pathogenesis and counteract host defense responses. Additionally, the enrichment of the term “structural molecule activity” is consistent with the activation of diverse genes involved in fungal growth. Given the presence of numerous genes with predicted functions associated with protein synthesis in *B. cinerea* during early infection of *S. lycopersicoides*, it is not surprising that the two most represented cellular components were “ribosome” and “protein complex”. Furthermore, representation of the term “mitochondrion” was enriched, as would be expected in a fungus displaying intense metabolic activity during pathogenesis.

For each *B. cinerea* gene identified, expression profiles were analyzed throughout the infection time course. Very few sequences from *B. cinerea* were detected at the 0 h time point (immediately after inoculation with fungal conidia), and thus expression of all fungal unigenes were significantly higher at 24 and 48 h after inoculation. Interestingly, genes implicated in pathogenesis and necrosis were abundantly expressed 24 and 48 h after inoculation, such as genes encoding an endopolygalacturonase (*Bcpg1*) demonstrated to play a role in virulence on tomato [4, 54], a superoxide dismutase (*bcsod1*) required for lesion expansion on *Phaseolus vulgaris*[55], and two cytochrome p450 monooxygenases (*BcBOT1* and *BcBOT2*) required for biosynthesis of the phytotoxin, botrydial [56, 57] (Additional file 3). The observed induction patterns of toxin biosynthetic genes, genes encoding cell wall degrading enzymes, and genes involved in scavenging reactive oxygen species indicate that *B. cinerea* actively induces necrosis in its host as early as 24 h after contact. In addition to genes involved in disease development, genes related to growth and energy production were among the most highly expressed in *B. cinerea* during pathogenesis, such as elongation factor 1 alpha and glyceraldehyde 3-phosphate dehydrogenase (Additional file 3). Although the primary objective of this study was to generate a sequence-based resource to identify genes in *S. lycopersicoides* involved in resistance to *B. cinerea*, the dataset created could assist efforts to identify novel genes in *B. cinerea* involved in early stages of infection.

## Discussion

Previous research has demonstrated that *S. lycopersicoides* is tolerant to abiotic stresses such as cold injury and nutrient deficiency, and is simultaneously resistant to diverse pathogens that are problematic on tomato, including viruses (tomato mosaic virus and cucumber mosaic virus), oomycetes (*Phytophthora parasitica*), and fungi (*Cladosporium fulvum* and *Botrytis cinerea*) [30, 31, 58]. In this study, *S. lycopersicoides* was confirmed to express resistance against *B. cinerea*, and newly found to be resistant to *A. solani. S. lycopersicoides* is a wild solanaceous species native to the Andean region of Chile and Peru, which is the center of diversity for many *Solanum* species [31, 58], and thus has likely evolved robust resistance responses to broad-range and host-specific necrotrophic fungal pathogens. Because *S. lycopersicoides* is closely related to and can be crossed with tomato [31], introgression lines have been created in which chromosomal segments from *S. lycopersicoides* have been incorporated into the genome of cultivated tomato [34]. Introgression lines provide a powerful resource for future determination of genes conferring resistance to *B. cinerea* and/or *A. solani*. Thus, genetic compatibility with cultivated tomato, a high level of resistance to necrotrophs, and availability of genetic resources make *S. lycopersicoides* an ideal source of novel genes to be harnessed through transgenic or conventional breeding techniques to improve the resistance of tomato to necrotrophic pathogens. By sequencing the transcriptome of *S. lycopersicoides* during early infection by *B. cinerea*, this work provides a novel and important resource for future work.

The molecular basis of resistance against *B. cinerea* and *A. solani* is not known. In general, plant resistance mechanisms to necrotrophic pathogens are believed to be distinct from or antagonistic to plant responses to biotrophs, which is consistent with their contrasting pathogenesis strategies [7, 8, 11]. Multiple examples signify differences in host resistance to these groups of pathogens [7, 59]. R-gene mediated resistance (e.g., effector triggered immunity, ETI) is normally activated upon recognition of race specific effector proteins by R-proteins and confers resistance to biotrophic pathogens [59]. ETI is a widespread and strong form of resistance but is not known to be effective against necrotrophs. Indeed, R-gene mediated susceptibility to necrotrophs has been documented [60–62]. The major manifestation of ETI is often the hypersensitive response (HR), a form of cell death, is central to plant resistance to biotrophs but promotes susceptibility to necrotrophs [6]. Production of reactive oxygen species (ROS) orchestrates HR and modulates resistance to biotrophs but may act as a virulence factor in some necrotrophs such as *B. cinerea*[63]. The signaling molecule salicylic acid (SA) promotes resistance to biotrophs but actually suppresses defense against necrotrophs [64, 65]. Systemic acquired resistance (SAR) is an SA-dependent resistance response that protects plants against many biotrophic pathogens [66–70] whereas its efficacy in conferring resistance to necrotrophs is unclear. Arabidopsis mutants impaired in SAR show normal resistance to necrotrophic fungi [67], whereas mutants that constitutively express SAR are more susceptible [71, 72]. Systemic and local defenses mediated by ethylene (ET) and jasmonate (JA) are required for resistance to necrotrophic pathogens [67, 73], whereas SA is generally associated with resistance to biotrophic infection [66, 69, 74, 75]*.* Although the scientific literature is replete with examples of antagonistic interactions between pathways mediated by SA and JA/ET in Arabidopsis, such interactions are not studied in other plant systems including tomato [7, 76–78]. These and many other examples suggest defense strategies that have evolved to guard plants against necrotrophs that operate distinctly or by antagonizing other responses.

The regulatory mechanism involved in host responses to broad-host necrotrophs such as *B. cinerea* is slowly emerging, predominantly from studies in Arabidopsis, but also to a limited extent in tomato. Diverse and unique processes that specifically mediate basal resistance to necrotrophs without any effect on biotrophic pathogens have been described. The tomato TPK1b and AIM1 function in defense against necrotrophic fungi with no role in resistance to other obligate or biotrophic pathogens [79, 80]. TPK1b function in defense is through modulation of ET signaling while AIM1 functions in ABA dependent immune responses. Many transcription-factors (TFs) that mediate defense response to necrotrophic infection have been identified through microarray and genetic analysis [46]. Among these, *WRKY33*, *ZFAR1*, *ERF1* and *ERF104,* MYB, AS1, and HD-Zip homeodomain proteins are required for resistance to necrotrophic fungi, underlining the importance of transcriptional regulation in defense to these pathogens [46, 81–86]. The role of transcriptional regulation is further reinforced by the recent discovery of the immune response functions of subunits of the transcriptional coactivator Mediator complex as specific regulators of plant immune responses to necrotrophs [87, 88]. Genetic evidence linking chromatin modifications such as histone ubiquitination, methylation, and deacetylation and chromatin remodeling to defense responses to necrotrophs due to their effects on expression of genes encoding various plant defense responses have been established [88–91]. Components of the plant cell wall and cuticle, predominantly considered physical barriers to infection, have found new and unexpected defense roles with mutants harboring defects in cuticle and cell wall components becoming more resistant to necrotrophs, thus revealing the dependence of virulence in necrotrophic fungi on critical host components [78, 92–96].

While the mechanisms of resistance to necrotrophic fungal pathogens are not fully understood, the ability of *S. lycopersicoides* to rapidly shift metabolism from photosynthesis to the production of resistance associated proteins and secondary metabolites appears to be a key factor for resistance to *B. cinerea*. Several classes of genes including pathogenesis related protein genes (PR1), protease genes (subtilisin) and glucanase genes (beta-1,3-glucanase) are rapidly and strongly induced in *S. lycopersicoides* in response to *B. cinerea* infection. However, this increased expression of defense related genes coincides with a reduced expression of genes involved in photorespiration such as ribulose-1,5-bisphosphate carboxylase and glycolate oxidase. This metabolic shunt occurs in *S. lycopersicum* as well as *S. lycopersicoides*, but at a slower rate and to less dramatic levels. Furthermore, metabolic pathway analysis in *S. lycopersicoides* demonstrates a shift within terpenoid biosynthesis away from the plastidic MEP pathway involved in pigment biosynthesis [97] to the mevalonate pathway involved in the synthesis of phytoalexins [98]. Taken together, these results point to a global change in metabolism that allows *S. lycopersicoides* to more effectively react to infection by necrotrophs.

In addition to identifying genes and metabolic changes associated with resistance to necrotrophs, this research has uncovered a number of fungal genes that are highly expressed during the early stages of infection of *S. lycopersicoides*. Several highly expressed genes, such as elongation factor 1 alpha and glyceraldehyde 3-phosphate dehydrogenase, are not surprising due to their fundamental roles in fungal growth. However, several genes coding hydrolytic enzymes, including an endo-polygalacturonase and an aspartic protease, as well as other genes, such as a cytochrome p450 monooxygenase required for the biosynthesis of phytotoxic secondary metabolites, were also induced. These findings demonstrate the potential value of the transcriptomic data generated in this research for identifying novel genes required for necrotrophy.

Another distinct value of this RNA-seq dataset is that it represents the first large-scale public sequence resource for *S. lycopersicoides*. Analogous to an EST sequencing experiment before the advent of next-generation sequencing, this study provides a dataset of species-specific sequence data for future validation of genome sequencing and identification of genes (based on homology as well as expression pattern) for functional characterization. Prior to this study, little information was available regarding molecular mechanisms of resistance in *S. lycopersicoides*. Based on the analyses of fungal growth and changes in host gene expression during the resistance response, a key mechanism of resistance appears to be constraining the growth of the pathogen through rapid and extensive reprogramming of the *S. lycopersicoides* transcriptome. In this study, numerous candidate defense-related genes were identified through clustering analyses; extensive functional characterization will be required to determine the genetic regulatory network underlying resistance.

It is important to note that RNA samples were pooled prior to sequencing in our approach, and thus the expression values obtained from sequencing the *S. lycopersicoides* transcriptome are indicative of qualitative trends in expression rather than exact quantitative measures of gene expression. Replicates were pooled to maximize the number of biological conditions evaluated within the experiment, and clustering analyses were performed to assess changes in expression. Sequencing separate replicates would have provided certain advantages, particularly with respect to calculating more precise digital expression values with greater rigor. However, pooled RNA samples are inherently normalized; expression is averaged among individuals, and thus this approach reduces the impact of isolated variability among individuals within a treatment. Similarly, pooled samples have proven useful to analyze differential expression in various other systems, including plants [99], animals [100, 101], and fungi [102, 103].

Research into mechanisms of plant resistance to necrotrophic fungal pathogens has been generally limited. A majority of studies, to date, have focused on Arabidopsis. Tomato, as a model for studying necrotrophic interactions, has been problematic due to the universal susceptibility of all tested varieties to important necrotrophs including *B. cinerea*. However, the availability of a resistant species that can be crossed with tomato provides a unique opportunity to study plant/necrotroph interactions in a commercially important crop species. Furthermore, the availability of this transcriptome data could be effectively used in conjunction with existing tomato lines containing defined introgressions of *S. lycopersicoides* chromosomal segments to identify features of the *S. lycopersicoides* genome that are crucial for resistance to necrotrophs.

## Conclusions

Tomato (*Solanum lycopersicum*), one of the world’s most important vegetable crops, is highly susceptible to necrotrophic fungal pathogens such as *Botrytis cinerea* and *Alternaria solani*. Improving resistance through conventional breeding has been hampered by a shortage of resistant germplasm and difficulties in introgressing resistance into elite germplasm without linkage drag. Screening of wild *Solanum* species uncovered a relative of tomato, *S. lycopersicoides*, that is resistant to both *B. cinerea* and *A. solani*. Transcriptome analysis of *S. lycopersicoides* at 0, 24, and 48 hours after inoculation with *B. cinerea* revealed possible mechanisms for resistance to necrotrophs and identified genes from *B. cinerea* that are induced during pathogenesis. Taken together, this research provides new insight into resistance to necrotrophs while providing a novel sequence resource for *S. lycopersicoides*.

## Methods

### Plant materials and fungal isolates

Accessions: LA0490 (*S. lycopersicum*, VF-36), LA2951 (*S. lycopersicoides*), LA3475 (*S. lycopersicum*, M-82), LA1932 (*S. chilense*), LA1708 (*S. arcanum*), LA1589 (*S. pimpinellifolium*), LA0716 (*S. pennellii*), LA0317 (*S. galapagense*), LA1777 (*S. habrochaites*), and LA1223 (*S. habrochaites f. glabratum*, Chimbalo) were developed by and/or obtained from the UC Davis/C.M. Rick Tomato Genetics Resource Center and maintained by the Department of Plant Sciences, University of California, Davis, CA 95616. *S. lycopersicum* cv. Bradley was obtained from the New England Seed Company (http://www.neseed.com/); Hartford, CT 06120). *S. lycopersicum* cv. Castlemart II was kindly provided by Greg Howe (Michigan State University). *B. cinerea* (B05.10) was maintained on 2xV8 agar in the dark at 25°C and *A. solani* (AR18, isolated from tomato in Arkansas) was maintained on V8 agar.

### Pathogen inoculation

Wild relatives of tomato and tomato cultivars were evaluated for their resistance to *B. cinerea* and *A. solani* by inoculating detached leaves. Inoculum of *B. cinerea* was prepared by cutting blocks of agar from 10-day-old cultures and agitating in 1% Sabouraud maltose broth (SMB). Conidia were separated from agar and mycelium by filtration through sterile cheesecloth. The spore concentration was checked with a hemacytometer and adjusted to 5×10^5^/ml with SMB. Detached leaves of *S. lycopersicum* and *S. lycopersicoides* (4 each per time point) were inoculated with 8 drops (5 μl each) of the *B. cinerea* spore suspension and placed on sterile filter paper moistened with sterile H_2_O in a covered petri dish. Inoculated leaves were incubated in a growth chamber with a 12/12 light/dark cycle at 21°C day and 18°C night temperatures. Lesion diameters were measured daily and a subset of leaves was collected each day for RNA extraction and ergosterol analysis. Due to low sporulation of the pathogen, mycelial fragments of *A. solani* at a concentration of 400 mg/mL was used for drop inoculation; otherwise conditions were similar to those described for *B. cinerea*.

### Quantification of ergosterol by HPLC

Inoculated leaves were frozen in liquid nitrogen and ground to a fine powder with a mortar and pestle. Ergosterol was then extracted from ground leaf tissue (150–550 mg) and analyzed by high pressure liquid chromatography as described by de Sio et al. [104] with minor adjustments. Briefly, ground leaves were added to 2.0 ml of 2:1 chloroform:methanol and extracted overnight. The extract was filtered through a 0.2 μm filter and 20 μl was injected onto a 25 mm C18 column (phenomenex, Torrance, CA). The mobile phase consisted of 80% methanol in H_2_O (solvent A) and 100% dichloromethane (solvent B). The gradient program consisted of a linear increase from 0% to 50% solvent B over 20 minutes followed by 15 minutes at 50% solvent B. Ergosterol was measured based on absorbance at 282 nm and was quantified based on comparison of peak area to pure standards (Alfa Aesar, Ward Hill, MA). Ergosterol concentration was then normalized to the mass of the extracted tissue and leaf mass.

### RNA extraction and cDNA synthesis

Inoculated leaves were frozen in liquid nitrogen and ground with a mortar and pestle. Total RNA was extracted from the ground tissue with TRIzol Reagent (Life Technologies, Grand Island, NY) according to the manufacturer’s instructions. RNA quantity and quality was determined with a NanoDrop spectrophotometer (Thermo Scientific, Wilmington, DE) and by visual inspection after electrophoresis. A total of 1 μg of RNA from each sample was treated with RQ1 DNase (Promega, Madison, WI) according to the manufacturer’s instructions. The DNase treated RNA (1 μg) was used as template to generate cDNA with M-MLV Reverse Transcriptase (Promega, Madison, WI) according to the manufacturer’s instructions.

### 454 sequencing and data processing

For transcriptome sequencing, *S. lycopersicoides* plants were spray inoculated with spores of *B. cinerea* at a concentration of 3×10^5^/ml. Total RNA was collected from inoculated leaves from 2 plants per time point at 0 hours, 24 hours, and 48 hours after inoculation. RNA from replicate leaf samples was pooled prior to sequencing. Conceptually, RNA pooling was performed as described by TJ Huth and SP Place [100], PA Olsvik, V Vikeså, KK Lie and EM Hevrøy [101] Library construction, sequencing, and *de novo* assembly were performed by the Purdue Genomics Core Facility (West Lafayette, IN). Read counts at each time point from individual isotigs within an isogroup were summed to reduce overrepresentation of genes with multiple splice variants. To identify unigenes from *S. lycopersicoides* and *B. cinerea*, as well as to remove contaminating sequences, Blast2GO (version 2.6.6) [105] was used to query the assembled unigenes against the nr database. The Audic and Claverie method [106] was used to identify plant unigenes that were differentially expressed between 0, 24, and 48 hours after inoculation with a false discovery rate of <0.0033. K-means clustering was performed on the differentially expressed plant genes with the genesis software (version 1.7.6) [107]. For K-means clustering, unigenes were assigned to one of four clusters. The basis for choosing four clusters was the closeness of fit of unigenes within each cluster, as well as the biological relevance of the expression patterns observed for each cluster. Blast2GO was used to functionally characterize unigenes within each plant cluster, as well as all fungal unigenes. InterProScan [108] was used to annotate unigenes with conserved protein domains. To identify GO terms that were enriched within each plant cluster, the Audic and Claverie method [106] was applied to all GO terms identified in all plant clusters. To make the number of GO terms associated with each cluster more manageable, GO slim analysis was performed with The Arabidopsis Information Resource (TAIR) GO slim for plants, while the Generic GO slim was applied to fungal unigenes.

### Analysis of gene expression with qPCR

cDNA from *S. lycopersicum* and *S. lycopersicoides* obtained immediately after (0 hours after inoculation), 24 hours after, or 48 hours after inoculation with *B. cinerea* was used as template for qPCR. qPCR was performed by combining SYBR green master mix (Life Technologies, Grand Island, NY) with primers (Additional file 4) and template according to the manufacturers instructions and monitoring fluorescence during template amplification in a stratagene M×300 P real-time PCR system (Agilent Technologies, Inc., Santa Clara, CA). The mean gene expression of three technical replications was normalized to expression of beta tubulin and calculated, relative to expression at 0 hours after inoculation, with the 2^-ΔΔCT^ method [109].

### Metabolic pathway analysis

Plant unigenes in each cluster were analyzed with the KEGG Automatic Annotation Server (KAAS) [110] to detect KEGG Orthologs (KO). KOs from clusters 1, 2, and 3 were combined into a single cluster representing up-regulated genes, while cluster 4 was kept separate to represent down-regulated genes. The KEGG Mapper – Reconstruct Pathway tool was then used to highlight genes within KEGG pathways that were up- or down-regulated in response to *B.cinerea*.

### Availability of supporting data

The 454 reads for *S. lycopersicoides* inoculated with *B. cinerea* have been submitted to NCBI sequence read archive (SRA, http://www.ncbi.nlm.nih.gov/sra) under the accession number SRR1054293.

## Electronic supplementary material

Additional file 1: **Excel file of expression and predicted identity of** ***S. lycopersicoides*** **unigenes.** (XLSX 96 KB)

Additional file 2: **KEGG analysis of the terpenoid backbone biosynthesis pathway in** ***Solanum lycopersicoides.*** The KEGG Automatic Annotation Service was used to assign KEGG Orthology (KO) identifiers to differentially expressed unigenes. The KO identifiers were then used to map unigenes to metabolic pathways. Genes highlighted in red indicate up-regulation in response to fungal infection. Genes highlighted in green indicate down-regulation in response to fungal infection. The pattern of gene expression indicates an activation of the mevalonate pathway in response to infection by *B. cinerea*. (TIFF 169 KB)

Additional file 3: **Excel file of expression and predicted identity of** ***B. cinerea*** **unigenes.** (XLSX 75 KB)

Additional file 4: **Excel file of primers used for analysis of gene expression by qPCR.** (XLSX 38 KB)

## References

[1] Divon HH, Fluhr R (2007). Nutrition acquisition strategies during fungal infection of plants. FEMS Microbiol Lett.

[2] Mendgen K, Hahn M (2002). Plant infection and the establishment of fungal biotrophy. Trends Plant Sci.

[3] Perfect SE, Green JR (2001). Infection structures of biotrophic and hemibiotrophic fungal plant pathogens. Mol Plant Pathol.

[4] van Kan JAL (2006). Licensed to kill: the lifestyle of a necrotrophic plant pathogen. Trends Plant Sci.

[5] Greenberg JT (1997). Programmed cell death in plant-pathogen interactions. Annu Rev Plant Physiol Plant Mol Biol.

[6] Govrin EM, Levine A (2000). The hypersensitive response facilitates plant infection by the necrotrophic pathogen Botrytis cinerea. Curr Biol.

[7] Glazebrook J (2005). Contrasting mechanisms of defense against biotrophic and necrotrophic pathogens. Annu Rev Phytopathol.

[8] Mengiste T (2012). Plant Immunity to Necrotrophs. Annu Rev Phytopathol.

[9] Schulze-Lefert P, Panstruga R (2003). Establishment of biotrophy by parasitic fungi and reprogramming of host cells for disease resistance. Annu Rev Phytopathol.

[10] Dodds PN, Rafiqi M, Gan PHP, Hardham AR, Jones DA, Ellis JG (2009). Effectors of biotrophic fungi and oomycetes: pathogenicity factors and triggers of host resistance. New Phytol.

[11] Laluk K, Mengiste T (2010). Necrotroph attacks on plants: wanton destruction or covert extortion?. Arabidopsis Book.

[12] Friesen TL, Faris JD, Solomon PS, Oliver RP (2008). Host-specific toxins: effectors of necrotrophic pathogenicity. Cell Microbiol.

[13] Jarvis WR (1977). Botryotinia and Botrytis species: taxonomy, physiology, and pathogenicity. Monograph, Research Branch Canada Department of Agriculture.

[14] **Genescope**. http://www.cns.fr/spip/Botrytis-cinerea-estimated-losses.html

[15] Colmenares AJ, Aleu J, Duran-Patron R, Collado IG, Hernandez-Galan R (2002). The putative role of botrydial and related metabolites in the infection mechanism of Botrytis cinerea. J Chem Ecol.

[16] Cutler HG, Parker SR, Ross SA, Crumley FG, Schreiner PR (1996). Homobotcinolide: a biologically active natural homolog of botcinolide from Botrytis cinerea. Biosci Biotechnol Biochem.

[17] Choquer M, Fournier E, Kunz C, Levis C, Pradier J-M, Simon A, Viaud M (2007). Botrytis cinerea virulence factors: new insights into a necrotrophic and polyphageous pathogen. FEMS Microbiol Lett.

[18] Williamson B, Tudzynski B, Tudzynski P, Van Kan JAL (2007). Botrytis cinerea: the cause of grey mould disease. Mol Plant Pathol.

[19] Tani H, Koshino H, Sakuno E, Nakajima H (2005). Botcinins A, B, C, and D, metabolites produced by Botrytis cinerea, and their antifungal activity against Magnaporthe grisea, a pathogen of rice blast disease. J Nat Prod.

[20] Tani H, Koshino H, Sakuno E, Cutler HG, Nakajima H (2006). Botcinins E and F and botcinolide from Botrytis cinerea and structural revision of botcinolides. J Nat Prod.

[21] Dalmais B, Schumacher J, Moraga J, Le Pecheur P, Tudzynski B, Gonzalez Collado I, Viaud M (2011). The Botrytis cinerea phytotoxin botcinic acid requires two polyketide synthases for production and has a redundant role in virulence with botrydial. Mol Plant Pathol.

[22] Shahbazi H, Aminian H, Sahebani N, Halterman D (2011). Effect of Alternaria solani exudates on resistant and susceptible potato cultivars from two different pathogen isolates. Plant Pathol J.

[23] Pound GS, Stahmann MA (1951). The production of a toxic material by Alternaria solani and its relation to the early blight disease of tomato. Phytopathology.

[24] Langsdorf G, Furuichi N, Doke N, Nishimura S (1990). Investigations on Alternaria solani infections: detection of alternaric acid and a susceptibility-inducing factor in the spore-germination fluid of A. solani. J Phytopathol.

[25] Ichihara A, Sakamura S, Tazaki H (1983). Solanapyrones A, B and C, phytotoxic metabolites from the fungus Alternaria solani. Tetrahedron Lett.

[26] Rigano MM, De Guzman G, Walmsley AM, Frusciante L, Barone A (2013). Production of pharmaceutical proteins in solanaceae food crops. Int J Mol Sci.

[27] ten Have A, van Berloo R, Lindhout P, van Kan JAL (2007). Partial stem and leaf resistance against the fungal pathogen Botrytis cinerea in wild relatives of tomato. Eur J Plant Pathol.

[28] Chaerani R, Groenwold R, Stam P, Voorrips RE (2007). Assessment of early blight (Alternaria solani) resistance in tomato using a droplet inoculation method. J Gen Plant Pathol.

[29] Gianessi L, Reigner N (2006). The importance of fungicides in U.S. crop production. Outlooks on Pest Management.

[30] Guimaraes RL, Chetelat RT, Stotz HU (2004). Resistance to Botrytis cinerea in Solanum lycopersicoides is dominant in hybrids with tomato, and involves induced hyphal death. Eur J Plant Pathol.

[31] Chetelat RT, Cisneros P, Stamova L, Rick CM (1997). A male-fertile Lycopersicon esculentum x Solanum lycopersicoides hybrid enables direct backcrossing to tomato at the diploid level. Euphytica.

[32] Finkers R, Bai Y, Berg P, Berloo R, Meijer-Dekens F, Have A, Kan J, Lindhout P, Heusden A (2008). Quantitative resistance to Botrytis cinerea from Solanum neorickii. Euphytica.

[33] Egashira H, Kuwashima A, Ishiguro H, Fukushima K, Kaya T, Imanishi S (2000). Screening of wild accessions resistant to grey mold (Botrytis cinerea Pers.) in Lycopersicon. Acta Physiol Plant.

[34] Canady MA, Meglic V, Chetelat RT (2005). A library of Solanum lycopersicoides introgression lines in cultivated tomato. Genome.

[35] Eshed Y, Zamir D (1995). An introgression line population of Lycopersicon pennellii in the cultivated tomato enables the identification and fine mapping of yield-associated QTL. Genetics.

[36] Blanca J, Cañzares J, Cordero L, Pascual L, Diez MJ, Nuez F (2012). Variation revealed by SNP genotyping and morphology provides insight into the origin of the tomato. PLoS One.

[37] Amselem J, Cuomo CA, van Kan JAL, Viaud M, Benito EP, Couloux A, Coutinho PM, de Vries RP, Dyer PS, Fillinger S, Fournier E, Gout L, Hahn M, Kohn L, Lapalu N, Plummer KM, Pradier J-M, Quévillon E, Sharon A, Simon A, ten Have A, Tudzynski B, Tudzynski P, Wincker P, Andrew M, Anthouard V, Beever RE, Beffa R, Benoit I, Bouzid O (2011). Genomic analysis of the necrotrophic fungal pathogens Sclerotinia sclerotiorum and Botrytis cinerea. PLoS Genet.

[38] Sato S, Tabata S, Hirakawa H, Asamizu E, Shirasawa K, Isobe S, Kaneko T, Nakamura Y, Shibata D, Aoki K, Egholm M, Knight J, Bogden R, Li C, Shuang Y, Xu X, Pan S, Cheng S, Liu X, Ren Y, Wang J, Albiero A, Dal Pero F, Todesco S, Van Eck J, Buels RM, Bombarely A, Gosselin JR, Huang M, Leto JA (2012). The tomato genome sequence provides insights into fleshy fruit evolution. Nature.

[39] Camon E, Magrane M, Barrell D, Lee V, Dimmer E, Maslen J, Binns D, Harte N, Lopez R, Apweiler R (2004). The Gene Ontology Annotation (GOA) database: sharing knowledge in uniprot with gene ontology. Nucleic Acids Res.

[40] Windram O, Madhou P, McHattie S, Hill C, Hickman R, Cooke E, Jenkins DJ, Penfold CA, Baxter L, Breeze E, Kiddle SJ, Rhodes J, Atwell S, Kliebenstein DJ, Kim Y-S, Stegle O, Borgwardt K, Zhang C, Tabrett A, Legaie R, Moore J, Finkenstadt B, Wild DL, Mead A, Rand D, Beynon J, Ott S, Buchanan-Wollaston V, Denby KJ (2012). Arabidopsis defense against Botrytis cinerea: chronology and regulation deciphered by high-resolution temporal transcriptomic analysis. Plant Cell.

[41] De Cremer K, Mathys J, Vos C, Froenicke L, Michelmore RW, Cammue BPA, De Coninck B (2013). RNAseq-based transcriptome analysis of Lactuca sativa infected by the fungal necrotroph Botrytis cinerea. Plant Cell Environ.

[42] Berger S, Papadopoulos M, Schreiber U, Kaiser W, Roitsch T (2004). Complex regulation of gene expression, photosynthesis and sugar levels by pathogen infection in tomato. Physiol Plant.

[43] Sánchez-Vallet A, López G, Ramos B, Delgado-Cerezo M, Riviere M-P, Llorente F, Fernández PV, Miedes E, Estevez JM, Grant M, Molina A (2012). Disruption of abscisic acid signaling constitutively activates Arabidopsis resistance to the necrotrophic fungus Plectosphaerella cucumerina. Plant Physiol.

[44] Loon LC, Rep M, Pieterse CMJ (2006). Significance of inducible defense-related proteins in infected plants. Annu Rev Phytopathol.

[45] Kamoun S (2006). A catalogue of the effector secretome of plant pathogenic oomycetes. Annu Rev Phytopathol.

[46] AbuQamar S, Chen X, Dhawan R, Bluhm B, Salmeron J, Lam S, Dietrich RA, Mengiste T (2006). Expression profiling and mutant analysis reveals complex regulatory networks involved in Arabidopsis response to Botrytis infection. Plant J.

[47] Marrs KA (1996). The functions and regulation of glutathione S-transferases in plants. Annu Rev Plant Physiol Plant Mol Biol.

[48] Bianchini GM, Paiva NL, Stermer BA (1996). Induction of early mevalonate pathway enzymes and biosynthesis of end products in potato (Solanum tuberosum) tubers by wounding and elicitation. Phytochemistry.

[49] Ha SH, Kim JB, Hwang YS, Lee SW (2003). Molecular characterization of three 3-hydroxy-3-methylglutaryl-CoA reductase genes including pathogen-induced Hmg2 from pepper (Capsicum annuum). Biochim Biophys Acta.

[50] Dubey VS, Bhalla R, Luthra R (2003). An overview of the non-mevalonate pathway for terpenoid biosynthesis in plants. J Biosci.

[51] Xiao Y, Savchenko T, Baidoo EEK, Chehab WE, Hayden DM, Tolstikov V, Corwin JA, Kliebenstein DJ, Keasling JD, Dehesh K (2012). Retrograde signaling by the plastidial metabolite MEcPP regulates expression of nuclear stress-response genes. Cell.

[52] Gil MJ, Coego A, Mauch-Mani B, Jordá L, Vera P (2005). The Arabidopsis csb3 mutant reveals a regulatory link between salicylic acid-mediated disease resistance and the methyl-erythritol 4-phosphate pathway. Plant J.

[53] Guest D, Brown J, Brown J, Ogle H (1997). Plant defences against pathogens. Plant pathogens and plant diseases.

[54] ten Have A, Mulder W, Visser J, van Kan JA (1998). The endopolygalacturonase gene Bcpg1 is required for full virulence of Botrytis cinerea. Mol Plant Microbe Interact.

[55] Rolke Y, Liu S, Quidde T, Williamson B, Schouten A, Weltring K-M, Siewers V, Tenberge KB, Tudzynski B, Tudzynski P (2004). Functional analysis of H (2) O (2)-generating systems in Botrytis cinerea: the major Cu-Zn-superoxide dismutase (BCSOD1) contributes to virulence on French bean, whereas a glucose oxidase (BCGOD1) is dispensable. Mol Plant Pathol.

[56] Pinedo C, Wang C-M, Pradier J-M, Dalmais B, Choquer M, Le Pêcheur P, Morgant G, Collado IG, Cane DE, Viaud M (2008). Sesquiterpene synthase from the botrydial biosynthetic gene cluster of the phytopathogen Botrytis cinerea. ACS Chem Biol.

[57] Wang C-M, Hopson R, Lin X, Cane DE (2009). Biosynthesis of the sesquiterpene botrydial in Botrytis cinerea. Mechanism and stereochemistry of the enzymatic formation of presilphiperfolan-8beta-ol. J Am Chem Soc.

[58] Zhao L, Qiu C, Li J, Chai Y, Kai G, Li Z, Sun X, Tang KX (2005). Investigation of disease resistance and cold tolerance of Solanum lycopersicoides for tomato improvement. HortSci.

[59] Jones JDG, Dangl JL (2006). The plant immune system. Nature.

[60] Lorang JM, Sweat TA, Wolpert TJ (2007). Plant disease susceptibility conferred by a “resistance” gene. Proc Natl Acad Sci U S A.

[61] Faris JD, Zhang Z, Lu H, Lu S, Reddy L, Cloutier S, Fellers JP, Meinhardt SW, Rasmussen JB, Xu SS, Oliver RP, Simons KJ, Friesen TL (2010). A unique wheat disease resistance-like gene governs effector-triggered susceptibility to necrotrophic pathogens. Proc Natl Acad Sci U S A.

[62] Nagy ED, Lee T-C, Ramakrishna W, Xu Z, Klein PE, SanMiguel P, Cheng C-P, Li J, Devos KM, Schertz K, Dunkle L, Bennetzen JL (2007). Fine mapping of the Pc locus of Sorghum bicolor, a gene controlling the reaction to a fungal pathogen and its host-selective toxin. Theor Appl Genet.

[63] Edlich W, Lorenz G, Lyr H, Nega E, Pommer EH (1989). New aspects on the infection mechanism of Botrytis cinerea Pers. Neth J Plant Pathol.

[64] Spoel SH, Johnson JS, Dong X (2007). Regulation of tradeoffs between plant defenses against pathogens with different lifestyles. Proc Natl Acad Sci U S A.

[65] Veronese P, Nakagami H, Bluhm B, AbuQamar S, Chen X, Salmeron J, Dietrich RA, Hirt H, Mengiste T (2006). The membrane-anchored BOTRYTIS-INDUCED KINASE1 plays distinct roles in Arabidopsis resistance to necrotrophic and biotrophic pathogens. Plant Cell.

[66] Delaney TP, Uknes S (1994). A central role of salicylic acid in plant disease resistance. Science.

[67] Thomma BP, Eggermont K, Penninckx IA, Mauch-Mani B, Vogelsang R, Cammue BP, Broekaert WF (1998). Separate jasmonate-dependent and salicylate-dependent defense-response pathways in Arabidopsis are essential for resistance to distinct microbial pathogens. Proc Natl Acad Sci U S A.

[68] Cao H, Glazebrook J, Clarke JD, Volko S, Dong X (1997). The Arabidopsis NPR1 gene that controls systemic acquired resistance encodes a novel protein containing ankyrin repeats. Cell.

[69] Reuber TL, Plotnikova JM, Dewdney J, Rogers EE, Wood W, Ausubel FM (1998). Correlation of defense gene induction defects with powdery mildew susceptibility in Arabidopsis enhanced disease susceptibility mutants. Plant J.

[70] Dempsey DA, Klessig DF, Shah J (1999). Salicylic acid and disease resistance in plants. Crit Rev Plant Sci.

[71] Kachroo P, Shanklin J, Shah J, Whittle EJ, Klessig DF (2001). A fatty acid desaturase modulates the activation of defense signaling pathways in plants. Proc Natl Acad Sci U S A.

[72] Govrin EM, Levine A (2002). Infection of Arabidopsis with a necrotrophic pathogen, Botrytis cinerea, elicits various defense responses but does not induce systemic acquired resistance (SAR). Plant Mol Biol.

[73] Thomma BP, Eggermont K, Tierens KF, Broekaert WF (1999). Requirement of functional ethylene-insensitive 2 gene for efficient resistance of Arabidopsis to infection by Botrytis cinerea. Plant Physiol.

[74] Métraux JP, Signer H, Ryals J, Ward E, Wyss-Benz M, Gaudin J, Raschdorf K, Schmid E, Blum W, Inverardi B (1990). Increase in salicylic Acid at the onset of systemic acquired resistance in cucumber. Science.

[75] Gaffney T, Friedrich L, Vernooij B, Negrotto D, Nye G, Uknes S, Ward E, Kessmann H, Ryals J (1993). Requirement of salicylic Acid for the induction of systemic acquired resistance. Science.

[76] Kunkel BN, Brooks DM (2002). Cross talk between signaling pathways in pathogen defense. Curr Opin Plant Biol.

[77] Anderson JP, Badruzsaufari E, Schenk PM, Manners JM, Desmond OJ, Ehlert C, Maclean DJ, Ebert PR, Kazan K (2004). Antagonistic interaction between abscisic acid and jasmonate-ethylene signaling pathways modulates defense gene expression and disease resistance in Arabidopsis. Plant Cell.

[78] Mang HG, Laluk KA, Parsons EP, Kosma DK, Cooper BR, Park HC, AbuQamar S, Boccongelli C, Miyazaki S, Consiglio F, Chilosi G, Bohnert HJ, Bressan RA, Mengiste T, Jenks MA (2009). The Arabidopsis RESURRECTION1 gene regulates a novel antagonistic interaction in plant defense to biotrophs and necrotrophs. Plant Physiol.

[79] Abuqamar S, Chai M-F, Luo H, Song F, Mengiste T (2008). Tomato protein kinase 1b mediates signaling of plant responses to necrotrophic fungi and insect herbivory. Plant Cell.

[80] Abuqamar S, Luo H, Laluk K, Mickelbart MV, Mengiste T (2009). Crosstalk between biotic and abiotic stress responses in tomato is mediated by the AIM1 transcription factor. Plant J.

[81] Zheng Z, Qamar SA, Chen Z, Mengiste T (2006). Arabidopsis WRKY33 transcription factor is required for resistance to necrotrophic fungal pathogens. Plant J.

[82] Mengiste T, Chen X, Salmeron J, Dietrich R (2003). The BOTRYTIS SUSCEPTIBLE1 gene encodes an R2R3MYB transcription factor protein that is required for biotic and abiotic stress responses in Arabidopsis. Plant Cell.

[83] Lorenzo O, Chico JM, Sánchez-Serrano JJ, Solano R (2004). JASMONATE-INSENSITIVE1 encodes a MYC transcription factor essential to discriminate between different jasmonate-regulated defense responses in Arabidopsis. Plant Cell.

[84] Bethke G, Unthan T, Uhrig JF, Pöschl Y, Gust AA, Scheel D, Lee J (2009). Flg22 regulates the release of an ethylene response factor substrate from MAP kinase 6 in Arabidopsis thaliana via ethylene signaling. Proc Natl Acad Sci U S A.

[85] Nurmberg PL, Knox KA, Yun B-W, Morris PC, Shafiei R, Hudson A, Loake GJ (2007). The developmental selector AS1 is an evolutionarily conserved regulator of the plant immune response. Proc Natl Acad Sci U S A.

[86] Coego A, Ramirez V, Gil MJ, Flors V, Mauch-Mani B, Vera P (2005). An Arabidopsis homeodomain transcription factor, OVEREXPRESSOR OF CATIONIC PEROXIDASE 3, mediates resistance to infection by necrotrophic pathogens. Plant Cell.

[87] Kidd BN, Edgar CI, Kumar KK, Aitken EA, Schenk PM, Manners JM, Kazan K (2009). The mediator complex subunit PFT1 is a key regulator of jasmonate-dependent defense in Arabidopsis. Plant Cell.

[88] Dhawan R, Luo H, Foerster AM, Abuqamar S, Du H-N, Briggs SD, Mittelsten Scheid O, Mengiste T (2009). HISTONE MONOUBIQUITINATION1 interacts with a subunit of the mediator complex and regulates defense against necrotrophic fungal pathogens in Arabidopsis. Plant Cell.

[89] Walley JW, Rowe HC, Xiao Y, Chehab EW, Kliebenstein DJ, Wagner D, Dehesh K (2008). The chromatin remodeler SPLAYED regulates specific stress signaling pathways. PLoS Pathog.

[90] Zhou C, Zhang L, Duan J, Miki B, Wu K (2005). HISTONE DEACETYLASE19 is involved in jasmonic acid and ethylene signaling of pathogen response in Arabidopsis. Plant Cell.

[91] Berr A, McCallum EJ, Alioua A, Heintz D, Heitz T, Shen W-H (2010). Arabidopsis histone methyltransferase SET DOMAIN GROUP8 mediates induction of the jasmonate/ethylene pathway genes in plant defense response to necrotrophic fungi. Plant Physiol.

[92] Cantu D, Vicente AR, Greve LC, Dewey FM, Bennett AB, Labavitch JM, Powell ALT (2008). The intersection between cell wall disassembly, ripening, and fruit susceptibility to Botrytis cinerea. Proc Natl Acad Sci U S A.

[93] Bessire M, Chassot C, Jacquat A-C, Humphry M, Borel S, Petétot JM-C, Métraux J-P, Nawrath C (2007). A permeable cuticle in Arabidopsis leads to a strong resistance to Botrytis cinerea. EMBO J.

[94] Chassot C, Nawrath C, Métraux J-P (2007). Cuticular defects lead to full immunity to a major plant pathogen. Plant J.

[95] Tang D, Simonich MT, Innes RW (2007). Mutations in LACS2, a long-chain acyl-coenzyme A synthetase, enhance susceptibility to avirulent Pseudomonas syringae but confer resistance to Botrytis cinerea in Arabidopsis. Plant Physiol.

[96] Hernández-Blanco C, Feng DX, Hu J, Sánchez-Vallet A, Deslandes L, Llorente F, Berrocal-Lobo M, Keller H, Barlet X, Sánchez-Rodríguez C, Anderson LK, Somerville S, Marco Y, Molina A (2007). Impairment of cellulose synthases required for Arabidopsis secondary cell wall formation enhances disease resistance. Plant Cell.

[97] Rohmer M (1999). The mevalonate-independent methylerythritol 4-phosphate (MEP) pathway for isoprenoid biosynthesis, including carotenoids. Pure Appl Chem.

[98] Stermer BA, Bianchini GM, Korth KL (1994). Regulation of HMG-CoA reductase activity in plants. J Lipid Res.

[99] Santos CS, Pinheiro M, Silva AI, Egas C, Vasconcelos MW (2012). Searching for resistance genes to Bursaphelenchus xylophilus using high throughput screening. BMC Genomics.

[100] Huth TJ, Place SP (2013). De novo assembly and characterization of tissue specific transcriptomes in the emerald notothen, Trematomus bernacchii. BMC Genomics.

[101] Olsvik PA, Vikeså V, Lie KK, Hevrøy EM (2013). Transcriptional responses to temperature and low oxygen stress in Atlantic salmon studied with next-generation sequencing technology. BMC Genomics.

[102] Ries L, Pullan ST, Delmas S, Malla S, Blythe MJ, Archer DB (2013). Genome-wide transcriptional response of Trichoderma reesei to lignocellulose using RNA sequencing and comparison with Aspergillus niger. BMC Genomics.

[103] Tremblay A, Hosseini P, Li S, Alkharouf NW, Matthews BF (2013). Analysis of Phakopsora pachyrhizi transcript abundance in critical pathways at four time-points during infection of a susceptible soybean cultivar using deep sequencing. BMC Genomics.

[104] de Sio F, Laratta B, Giovane A, Quagliuolo L, Castaldo D, Servillo L (2000). Analysis of free and esterified ergosterol in tomato products. J Agric Food Chem.

[105] Conesa A, Götz S, García-Gómez JM, Terol J, Talón M, Robles M (2005). Blast2GO: a universal tool for annotation, visualization and analysis in functional genomics research. Bioinformatics.

[106] Audic S, Claverie JM (1997). The significance of digital gene expression profiles. Genome Res.

[107] Sturn A, Quackenbush J, Trajanoski Z (2002). Genesis: cluster analysis of microarray data. Bioinformatics.

[108] Zdobnov EM, Apweiler R (2001). InterProScan--an integration platform for the signature-recognition methods in InterPro. Bioinformatics.

[109] Livak KJ, Schmittgen TD (2001). Analysis of relative gene expression data using real-time quantitative PCR and the 2(−Delta Delta C (T)) Method. Methods.

[110] Moriya Y, Itoh M, Okuda S, Yoshizawa AC, Kanehisa M (2007). KAAS: an automatic genome annotation and pathway reconstruction server. Nucleic Acids Res.

