# Supplementary figures and images for: Resistance to Botrytis cinerea in Solanum lycopersicoides involves widespread transcriptional reprogramming
Source: BMC Genomics. 2014 May 3;15:334. doi: 10.1186/1471-2164-15-334 (PMC4035065; doi:10.1186/1471-2164-15-334)

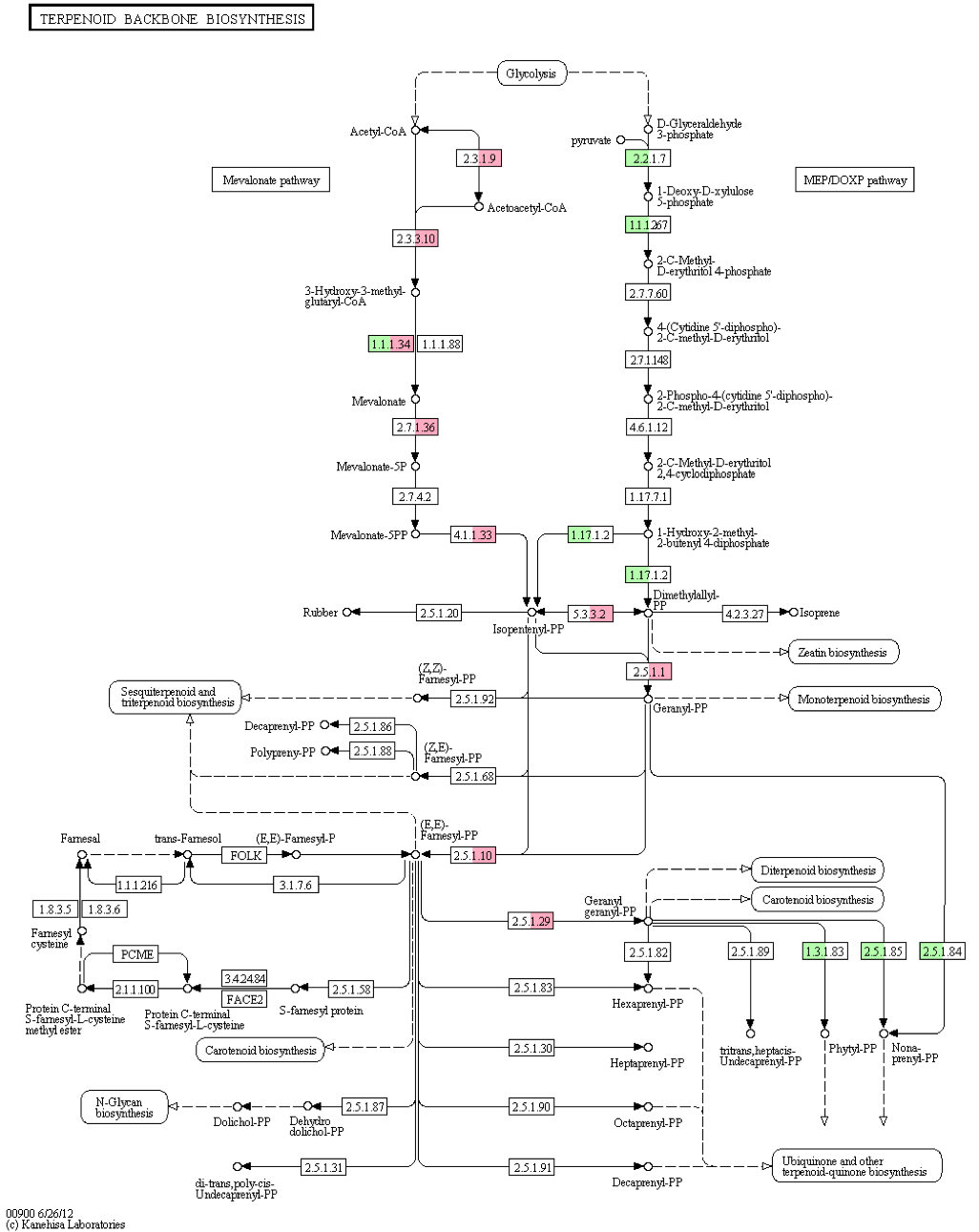

Supplement: Supplementary file 2 — Additional file 2: KEGG analysis of the terpenoid backbone biosynthesis pathway in Solanum lycopersicoides. The KEGG Automatic Annotation Service was used to assign KEGG Orthology (KO) identifiers to differentially expressed unigenes. The KO identifiers were then used to map unigenes to metabolic pathways. Genes highlighted in red indicate up-regulation in response to fungal infection. Genes highlighted in green indicate down-regulation in response to fungal infection. The pattern of gene expression indicates an activation of the mevalonate pathway in response to infection by B. cinerea. (TIFF 169 KB) [file 12864_2013_6020_MOESM2_ESM.tiff]
